# Supplementary material for: Effect of storage conditions on SARS-CoV-2 RNA quantification in wastewater solids
Source: PeerJ. 2021 Aug 11;9:e11933. doi: 10.7717/peerj.11933 (PMC8364322; doi:10.7717/peerj.11933)
Supplement: Supplemental Information 1 — Details pertaining to the minimal data for reporting digital PCR data are included in this file (MIQE guidelines) [file peerj-09-11933-s001.docx]

Supporting material

The average (standard deviation) number of droplets in ten merged wells determined from a random subset of 8 samples was 190000 (6800). Average number of copies per partition (λ) for SARS-CoV-2 RNA in the same subset was 7.2x10^-4^, the average of the number of copies per partition (λ) for PMMoV was 0.14, and 1.8x10^-3^ for BCoV.

As the samples were extracted ten times and each extract analyzed in one of 10 replicate wells which were merged, the replicate variability incorporates variation from both RNA extraction and RT-dPCR with a heterogeneous solids sample. Sample standard deviations for the SARS-CoV-2, PMMoV RNA, and BCoV RNA quantification estimated from the merged wells were, on average 21%, 17%, and 15% of the measurement. Assays were conducted in only one lab, so reproducibility was not assessed.

The LOD of the assay was 3 positive droplets which translates into between 600 and 1000 copies/g dry weight depending on the percent dry weight of the solids used in the extraction which depends on the properties of the solid and how effectively it can be dewatered.

Example fluorescence plots for the N, S, and ORF1a genes are provided in the associated reference by Topol et al. [1], additional fluorescence plots are shown below in Figures S1, S2, and S3.

Additional MIQE guideline information is provided in Table S1. The references in the table are provided and numbered according to this Supporting Material document.

Captions for all Figures and are provided with the associated file in the supporting material.

|  |
| --- |

References

[1] [Topol A, Wolfe M, White B, Wigginton K, Boehm A. High Throughput SARS-COV-2, PMMOV, and BCoV quantification in settled solids using digital RT-PCR. protocols.io. 2021. Available: dx.doi.org/10.17504/protocols.io.btywnpxe](https://www.zotero.org/google-docs/?4ha0mu)

[2] [The dMIQE Group, Huggett JF. The Digital MIQE Guidelines Update: Minimum Information for Publication of Quantitative Digital PCR Experiments for 2020. Clinical Chemistry. 2020;66: 1012–1029. doi:10.1093/clinchem/hvaa125](https://www.zotero.org/google-docs/?4ha0mu)

[3] Huisman JS, Scire J, Caduff L, Fernandez-Cassi X, Ganesanandamoorthy P, Kull A, Andreas Scheidegger, Elyse Stachler, Alexandria B. Boehm, Bridgette Hughes, Alisha Knudson, Aaron Topol, Krista R. Wigginton, Marlene K. Wolfe, Tamar Kohn, Christoph Ort, Tanja Stadler, Timothy R. Julian. Wastewater-based estimation of the effective reproductive number of SARS-CoV-2. medRxiv. 2021; 2021.04.29.21255961. doi:10.1101/2021.04.29.21255961
