# Supplementary material for: Effect of storage conditions on SARS-CoV-2 RNA quantification in wastewater solids
Source: PeerJ. 2021 Aug 11;9:e11933. doi: 10.7717/peerj.11933 (PMC8364322; doi:10.7717/peerj.11933)
Supplement: Supplemental Information 2 — Authors should fill detail whether information is provided. Where ‘yes’ is selected use comment box to detail location of information or to include the information. Where ‘no’ is selected use comment box to outline rationale for omission. Sections 4 and 5 may not apply depending on experiment. From Graham et al. (2021). Peccia et al. (2020) is cited in the table. [file peerj-09-11933-s002.pdf]

ITEM TO CHECK	PROVIDED	COMMENT
	Y/N	
**1. SPECIMEN**		
Detailed description of specimen type and numbers	Y	Methods of Main Text
Sampling procedure (including time to storage)	Y	Methods of Main Text
Sample aliquotation, storage conditions and duration	Y	Methods of Main Text
**2. NUCLEIC ACID EXTRACTION**		
Description of extraction method including amount of sample processed	Y	Methods of Main Text
Volume of solvent used to elute/resuspend extract	Y	Methods of Main Text
Number of extraction replicates	Y	Methods of Main Text
Extraction blanks included?	Y	Methods of Main Text
**3. NUCLEIC ACID ASSESSMENT AND STORAGE**		
Method to evaluate quality of nucleic acids	N	Not Done
Method to evaluate quantity of nucleic acids (including molecular weight	N	Not Done
Storage conditions: temperature, concentration, duration, buffer, aliquots	Y	Methods of Main Text
Clear description of dilution steps used to prepare working DNA solution	Y	Methods of Main Text
**4. NUCLEIC ACID MODIFICATION**		
Template modification (digestion, sonication, pre-amplification,	N	NA
Details of repurification following modification if performed	Y	Zymo Column in Methods of Main Text
**5. REVERSE TRANSCRIPTION**		
cDNA priming method and concentration	N	NA
One or two step protocol (include reaction details for two step)	Y	Methods of Main Text
Amount of RNA added per reaction	Y	Methods of Main Text
Detailed reaction components and conditions	Y	Methods of Main Text, also provided in protocols.io protocol referenced in main text
Estimated copies measured with and without addition of RT*	N	Not Done
Manufacturer of reagents used with catalogue and lot numbers	N	Reagents, Manufacturers, and Catalogue Numbers Reported in Supplemental Material. Lot Numbers are Not Reported
Storage of cDNA: temperature, concentration, duration, buffer and	Y	NA
**6. dPCR OLIGONUCLEOTIDES DESIGN AND TARGET INFORMATION**		
Sequence accession number or official gene symbol	Y	Provided in reference in paper to Huisman et al.
Method (software) used for design and *in silico* verification	Y	Provided in reference in paper to Huisman et al.
Location of amplicon	Y	Provided in reference in paper to Huisman et al.
Amplicon length	Y	Provided in reference in paper to Huisman et al.
Primer and probe sequences (or amplicon context sequence)**	Y	Methods in Main Text
Location and identity of any modifications	Y	NA
Manufacturer of oligonucleotides	Y	Methods in Main Text
**7. dPCR PROTOCOL**		
Manufacturer of dPCR instrument and instrument model	Y	Methods of Main Text, also provided in protocols.io protocol
Buffer/kit manufacturer with catalogue and lot number	Y	Methods of Main Text, also provided in protocols.io protocol
Primer and probe concentration	Y	Methods of Main Text, also provided in protocols.io protocol
Pre-reaction volume and composition (incl. amount of template and if	Y	Methods of Main Text, also provided in protocols.io protocol
Template treatment (initial heating or chemical denaturation)	N	NA
Polymerase identity and concentration, Mg++ and dNTP concentrations***	N	Included in Kit Manuals
Complete thermocycling parameters	Y	Methods of Main Text, also provided in protocols.io protocol referenced in main text
**8. ASSAY VALIDATION**		
Details of optimisation performed	N	Commercial Kit, Followed Manufacturer's Instructions
Analytical specificity (vs. related sequences) and limit of blank (LOB)	N	Provided in reference in paper to Huisman et al.
Analytical sensitivity/LoD and how this was evaluated	Y	Methods of Main Text
Testing for inhibitors (from biological matrix/extraction)	Y	Methods of Main Text
**9. DATA ANALYSIS**		
Description of dPCR experimental design	Y	Methods of Main Text
Comprehensive details negative and positive of controls (whether applied	Y	Methods of Main Text
Partition classification method (thresholding)	Y	Methods of Main Text, also provided in protocols.io protocol referenced in main text
Examples of positive and negative experimental results (including	Y	Supplemental Information
Description of technical replication	Y	Methods of Main Text
Repeatability (intra-experiment variation)	Y	Supplemental Information
Reproducibility (inter-experiment/user/lab etc. variation)	N	Assays were only completed within one laboratory
Number of partitions measured (average and standard deviation)	Y	Supplemental Information
Partition volume	Y	Reported by Manufacturer
Copies per partition (λ or equivalent) (average and standard deviation)	Y	Supplemental Information
dPCR analysis program (source, version)	Y	Methods of Main Text
Description of normalisation method	Y	Methods of Main text when applicable
Statistical methods used for analysis	Y	Methods of Main Text
Data transparency	raw data uploaded to online repository with ID:	https://purl.stanford.edu/yn042kx5009
