# Supplementary figures and images for: Effect of storage conditions on SARS-CoV-2 RNA quantification in wastewater solids
Source: PeerJ. 2021 Aug 11;9:e11933. doi: 10.7717/peerj.11933 (PMC8364322; doi:10.7717/peerj.11933)

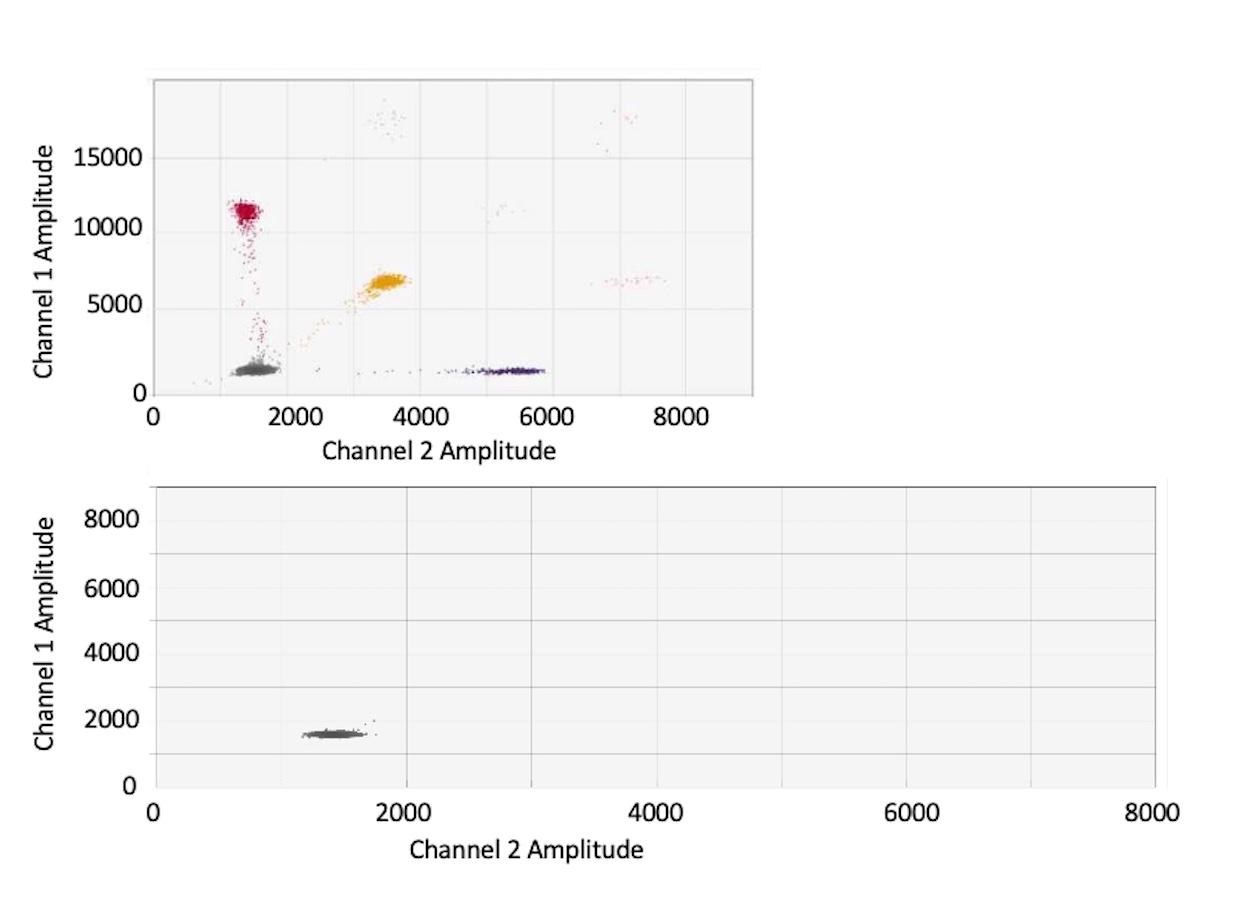

Supplement: Supplemental Information 3 — Positive experimental results are provided in the top image and negative experimental results are shown in the bottom image. [file peerj-09-11933-s003.jpg]

Amplitude

10000

8000

6000

4000

2000

0

BCoV 1:100

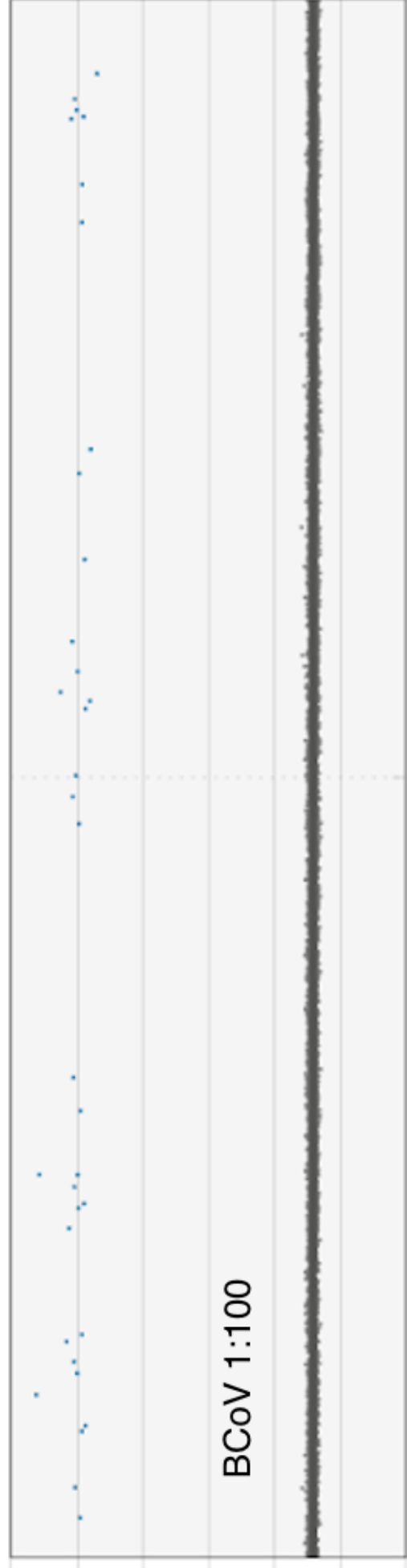

Amplitude

3000

2000

1000

0

PMMoV 1:100

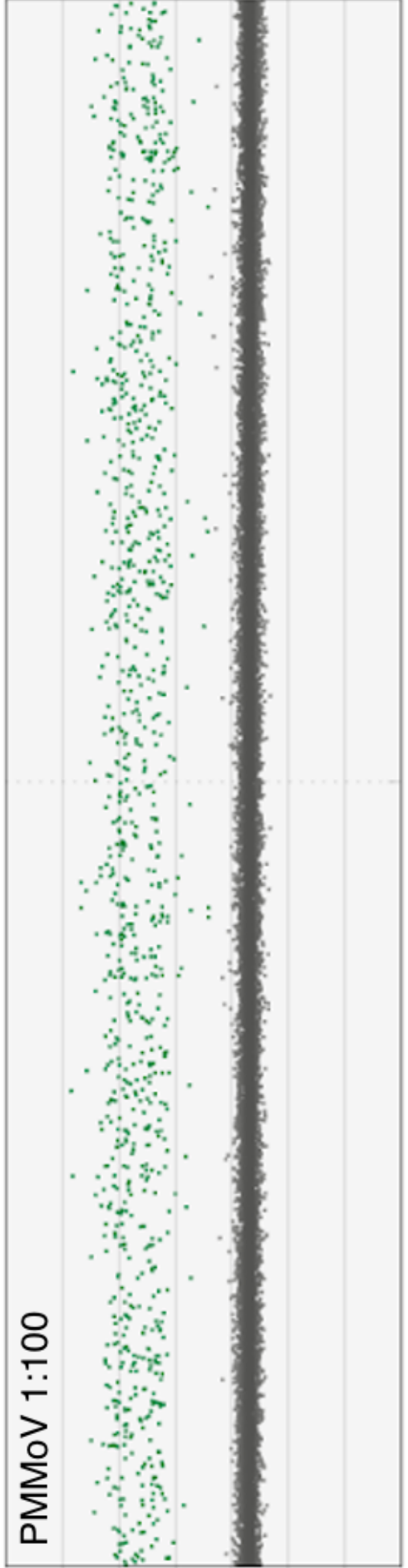

Supplement: Supplemental Information 4 — Positive experimental results are shown. [file peerj-09-11933-s004.pdf]
