# Supplementary material for: Effect of storage conditions on SARS-CoV-2 RNA quantification in wastewater solids
Source: PeerJ. 2021 Aug 11;9:e11933. doi: 10.7717/peerj.11933 (PMC8364322; doi:10.7717/peerj.11933)
Supplement: Supplemental Information 5 — Negative experimental results are shown. [file peerj-09-11933-s005.pdf]

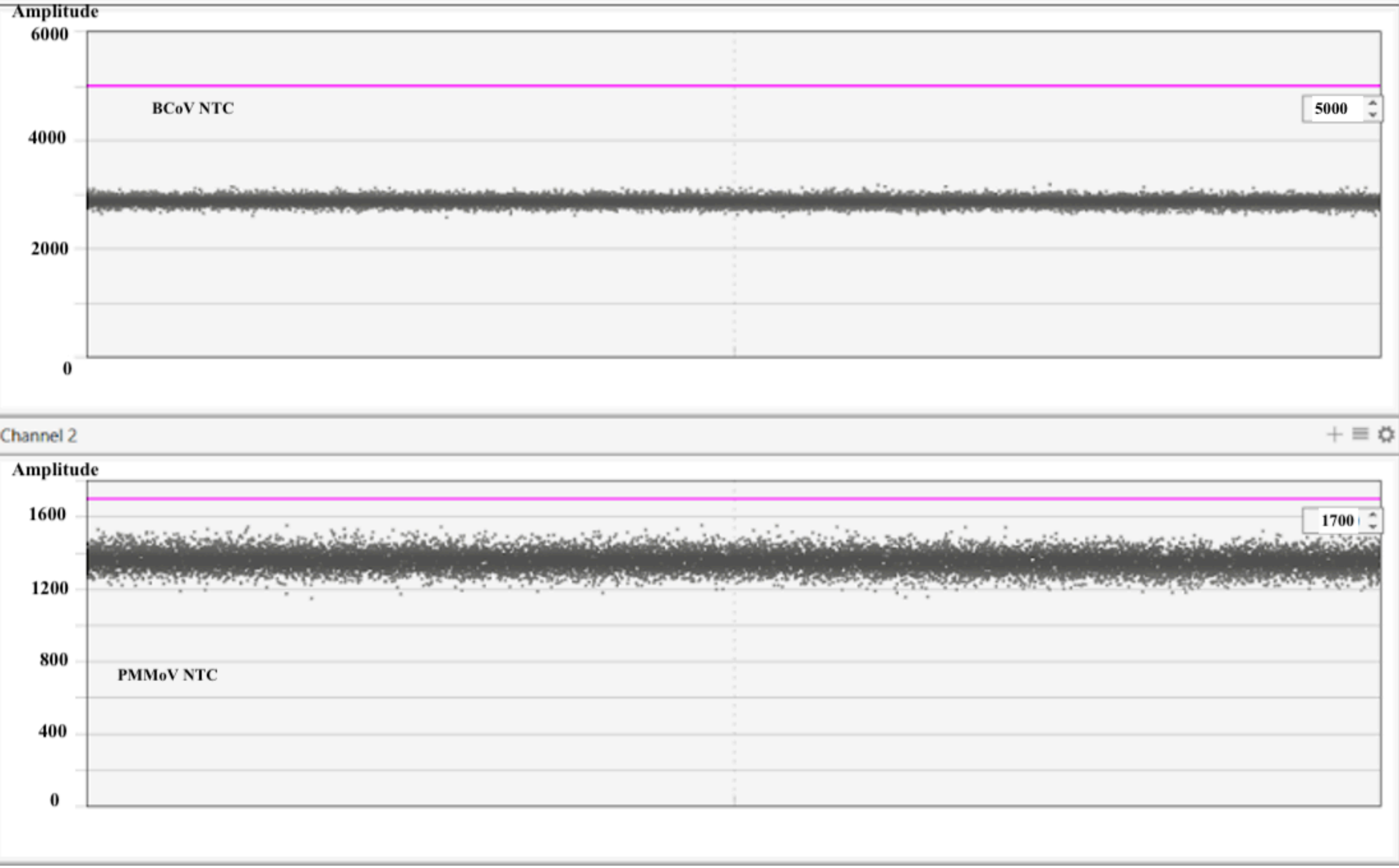
Amplitude
6000
4000
2000
0
BCoV NTC
5000
Channel 2
Amplitude
1600
1200
800
400
0
PMMoV NTC
1700
